# The Discovery of Abl Kinase ATPase Activity and Its Implications in the Development of Straightforward Assays

**DOI:** 10.1021/acsomega.5c08176

**Published:** 2025-11-12

**Authors:** Diego Magno Martins, Philipe Oliveira Fernandes, Lucas Almeida Vieira, Tiago Antônio Brandão, Adolfo Henrique Moraes

**Affiliations:** † Departamento de Química, Instituto de Ciências Exatas, 28114 Universidade Federal de Minas Gerais (UFMG), 31270-901 Belo Horizonte, MG, Brazil; ‡ Departamento de Produtos Farmacêuticos, Faculdade de Farmácia, Universidade Federal de Minas Gerais (UFMG), 31270-901 Belo Horizonte, MG, Brazil; § Laboratório de Ressonância Magnética de Alta Resolução, Universidade Federal de Minas Gerais (UFMG), 31270-901 Belo Horizonte, MG, Brazil

## Abstract

Abelson kinase (Abl) is an enzyme crucial in metabolic pathways, and it is a molecular target for leukemias, especially chronic myeloid leukemia. Despite extensive research on its kinase function and inhibition over the years, there is still considerable room for new discoveries regarding its mechanistic and reactive aspects. We report here, for the first time, the intrinsic ATPase activity of Abl in the absence of peptide substrates. Using quantitative one-dimensional ^31^P nuclear magnetic resonance spectroscopy, we monitored the conversion of ATP to ADP and inorganic phosphate (Pi) catalyzed by Abl. Furthermore, we demonstrated that the known kinase inhibitors imatinib and dasatinib inhibit ATPase activity. Beyond expanding the biochemical repertoire of Abl, this finding enables a straightforward, substrate-free assay for kinase inhibition, offering new perspectives on kinase catalytic plasticity and laying the groundwork for simplified screening strategies in drug discovery.

## Graphical abstract

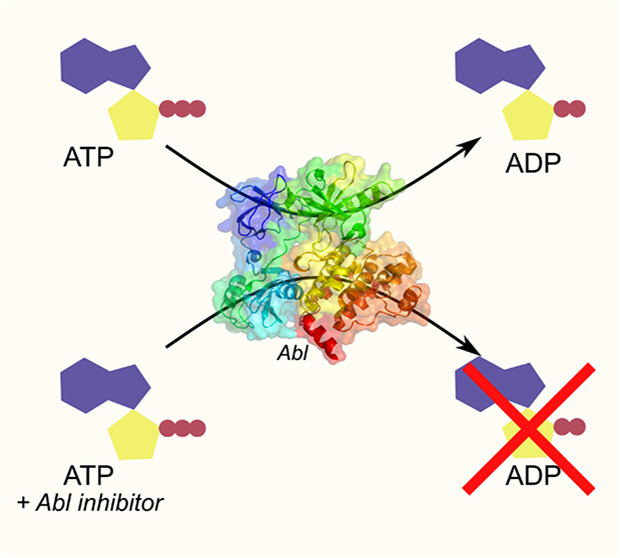

## 1 Introduction

Protein kinases are central regulators of cellular signaling pathways; phosphoproteomic investigations indicate that the majority of proteins expressed in mammalian cells are subject to phosphorylation.^1^ Abelson kinase (Abl) is a well-studied human tyrosine kinase that plays a crucial role in cellular processes involved in DNA repair, cytoskeletal dynamics, and cellular apoptosis.^2–7^ A dysregulation of these processes is related to different diseases, such as chronic myeloid leukemia (CML) and acute lymphoblastic leukemia (ALL).^8,9^ Abl kinase intrigues researchers due to its multifaceted roles in cellular processes. Its modular architecture, comprising a kinase domain and regulatory SH2/SH3 domains, has been extensively studied. Conformational states, autoinhibition mechanisms, and ligand-induced activation have been resolved.^10–16^

Moreover, structural investigations have provided insights into the therapeutic mechanisms by revealing the binding modes of small-molecule inhibitors, such as imatinib and dasatinib, as well as the consequences of the binding process.^17^ Despite numerous studies on Abl and other tyrosine kinases, many aspects of their function, regulation, and interactions remain to be discovered. One example of a noncanonical activity of protein kinases is their potential ability to hydrolyze ATP—referred to as ATPase activity.

Here, we describe for the first time the intrinsic ATPase activity of full-length Abl kinase by tracking the conversion of ATP into ADP and inorganic phosphate (Pi) in the absence of any additional substrate. These findings add a new dimension to understanding its enzymatic repertoire, providing novel insights into its biochemical and cellular roles. Furthermore, we demonstrate that this activity can be leveraged to assess kinase inhibition by applying this method to CML-approved drugs.

## 2 Materials and Methods

### 2.1 ^31^P Quantitative NMR Experiments

To determine the phosphate concentration in solution, we created a calibration curve using seven solutions containing dibasic potassium phosphate (K_2_HPO_4_) concentrations ranging from 0 to 2 mM, performed in triplicate. The samples were prepared in a Tris-HCl 40 mM, pH 7.5 buffer, 2.5 mM MgCl_2_, 2% dimethyl sulfoxide (DMSO) (v/v), 11,7% D_2_O (v/v), and the K_2_HPO_4_ salt was prepared as described by Morita and Assumpção.^18^ The reaction in the presence of Abl was quenched by adding ethylenediaminetetraacetic acid (EDTA, 300 mM, pH 13) to a final concentration of 30 mM. Trimethyl phosphate (TMP) was used as an internal standard for quantification at a final concentration of 2.14 mM. The NMR experiments were conducted using a Bruker Avance III nanobay 400 MHz with a PABBFO-H-D/Z-grad 5 mm probe. The spectra were acquired with 65,536 points, a spectral window of 99.79 ppm, and 900 scans at 25 °C. Furthermore, a 60° pulse and a recovery time (D1) of 7 s were employed. The standard curve was determined by measuring the ^31^P NMR signal integral ratio of K_2_HPO_4_ to TMP (*A*_Pi_/*A*_TMP_) and plotted against the K_2_HPO_4_ concentration. Lastly, the spectra were processed and analyzed using Topspin 4.3.0 software (Bruker Corporation, USA).

Least-squares fitting was applied to the data and the coefficient of determination. The limits of detection (LOD) and quantification (LOQ) were determined based on calibration curves. Noise integration in the NMR spectra was performed over a 0.4 ppm range, matching the width used to integrate the phosphate signal in the calibration curve samples. The LOD and LOQ were then estimated using 20 noise regions in the NMR spectra. These values were obtained following standard methodology, where LOD is defined as 3.3 times the standard deviation of the noise (σ) divided by the slope (*S*) of the calibration curve (LOD = 3.3σ/*S*), and LOQ is defined as ten times the standard deviation of the noise divided by the slope (LOQ = 10σ/*S*).^19,20^

### 2.2 Abl Kinase ATPase Activity Evaluation

Abl enzymatic reactions were performed at 20 °C in a reaction buffer with 40 mM Tris-HCl (pH 7.5), 2.5 mM magnesium chloride, 2% DMSO, and 11.7% D_2_O. After dilution, the final D_2_O concentration was 10%, allowing for frequency lock in NMR experiments. Reactions were initiated by adding ATP to a final concentration of 1.6 mM. The concentration of Abl kinase varied between 0 and 1.53 μM, depending on the aim of each experiment (further details on protein expression and purification are described in the Supporting Information). The final reaction volume was 605 μL. All reactions were quenched by adding the stop solution, an EDTA solution (300 mM, pH 13), to a final concentration of 30 mM. The TMP standard was also added to a final concentration of 2.14 mM. To evaluate the sensitivity of the Abl ATPase activity to the addition of known Abl kinase inhibitors, assays with imatinib and dasatinib were performed. Imatinib concentrations varied from 0 to 20 μM, while dasatinib was used at a fixed concentration of 3 μM. The enzyme was incubated for 10 min in the buffered solution with the inhibitors before ATP addition, and the reaction was stopped after 120 min.

### 2.3 Model Fitting and Analysis

The Pi concentration was estimated by applying the standard curve described in the section 2.1, and the time-course data were used to calculate the rate of phosphate production, which reflects ATP hydrolysis by Abl kinase. The enzymatic activity was expressed as *k*_cat_, in min^–1^, according to the following equation:

1 $$k_{\mathrm{cat}}=\frac{{\Delta C}_{\mathrm{prod}}/\Delta t}{{\left[E\right]}_{T}}$$

where Δ*C*_prod_ is the change in phosphate concentration, Δ*t* is the time interval, and [E]_T_ is the enzyme concentration.

The percentage of inhibition (Inhibition %) of each inhibitor’s concentration was calculated using the following equation:

2 $$\mathrm{Inhibition}\%=\frac{C_{\mathrm{prod}}\left(\mathrm{without}\;\mathrm{inhibitor}\right)-C_{\mathrm{prod}}\left(\mathrm{with}\;\mathrm{inhibitor}\right)}{C_{\mathrm{prod}}\left(\mathrm{without}\;\mathrm{inhibitor}\right)}\cdot 100$$

where *C*_prod_(without inhibitor) is the phosphate concentration in the reaction without inhibitor and *C*_prod_(with inhibitor) is the phosphate concentration in the presence of inhibitors.

The apparent dissociation constant (*K*_d_^ap^) for imatinib binding to Abl was determined from the percentage of inhibition (Inhibition %) as a function of the total inhibitor concentration ([I]_T_). Data were acquired as percent inhibition versus [I]_T_ at fixed enzyme and ATP concentrations ([E]_T_ and [ATP], respectively). The analysis assumed a 1:1 binding stoichiometry (E + I ⇌ EI), in which the EI complex is catalytically inactive.

Because the ATPase reaction is a secondary process, a relatively high enzyme concentration ([E]_T_ = 1.27 μM) was required to yield a detectable inorganic phosphate signal by ^31^P NMR within a reasonable experimental time frame. However, such high enzyme concentration invalidates the use of the classical Hill or Michaelis–Menten approximations, since the assumption [E]_T_ ≪ *K*_d_ no longer holds. Under these conditions, known as tight-binding inhibition, the free inhibitor concentration is substantially depleted upon enzyme binding, and a correction for this effect becomes mandatory. Therefore, the inhibition data were analyzed using the Morrison equation,^21^ which explicitly accounts for the stoichiometric binding of inhibitors by the enzyme.^22,23^

At equilibrium, the fraction of enzyme present as EI (Frac_inh_) is expressed by the Morrison tight-binding equation using total concentrations

3 $${\mathrm{Frac}}_{\mathrm{in}h}\left({\left[I\right]}_{T}\right)=\{{\left[E\right]}_{T}+{\left[I\right]}_{T}+K_{d}^{\mathrm{app}}-\sqrt{{\left({\left[E\right]}_{T}+{\left[I\right]}_{T}+K_{d}^{\mathrm{app}}\right)}^{2}-4{\left[E\right]}_{T}{\left[I\right]}_{T}}\}/2{\left[E\right]}_{T}$$

where [E]_T_ and [I]_T_ are the total enzyme and inhibitor concentrations, respectively, and *K*_d_^app^ is the apparent dissociation constant under the specific assay conditions.

The observed percentage of inhibition was modeled as

4 $$\mathrm{In}h\mathrm{ibition}\left({\left[I\right]}_{T}\right)\left(\%\right)=I_{\min}+\left(I_{\max}-I_{\min}\right)\times {\mathrm{Frac}}_{\mathrm{in}h}\left({\left[I\right]}_{T}\right)$$

where *I*_min_ and *I*_max_ represent the lower and upper asymptotic limits of inhibition (typically 0% and 100%, respectively), accounting for potential baseline drift or incomplete inhibition.

For a competitive inhibitor in the presence of ATP, the apparent dissociation constant (*K*_d_^app^) relates to the intrinsic dissociation constant (*K*_d_) through a Cheng–Prusoff-like correction.^24,25^

5 $$K_{d}=\frac{K_{d}^{\mathrm{app}}}{1+\frac{\left[\mathrm{ATP}\right]}{K_{m}}}$$

where [ATP] is the ATP concentration and *K*_m_ the Michaelis constant for ATP binding to Abl. In this study, an ATP *K*_m_ value of 43.6 μM, previously reported for Abl,^26^ was used to convert *K*_d_^app^ to the intrinsic *K*_d_.

Experimental inhibition data were fitted by nonlinear least-squares regression using the tight-binding model described above (eqs 3–5). The fitting parameters were *K*_d_^app^, *I*_min_, and *I*_max_, with the total enzyme concentration [E]_T_, and ATP *K*_m_ of binding to Abl fixed at their nominal value. Confidence intervals (95%) were estimated from the covariance matrix of the fit. All data processing and regression analyses were performed using custom Python 3 scripts with SciPy 1.10.1 and NumPy 1.26 libraries, Microsoft Excel Office 2019, and OriginPro 2018.

## 3 Results and Discussion

### 3.1 Phosphate Formation Quantification by ^31^P Quantitative NMR Experiments

^31^P NMR is a robust and well-established method for quantifying inorganic phosphate (Pi), offering specificity and accuracy. The selectivity of ^31^P NMR eliminates signal overlap from other phosphorus-containing molecules, ensuring high-precision analysis of phosphate concentrations. This method has been extensively validated in the literature^27–29^ and is widely used to study phosphate metabolism,^30,31^ enzymatic reactions involving ATP,^32^ and phosphate transport kinetics.^33,34^ Its high sensitivity, combined with the absence of overlapping signals from other phosphorus species, ensures reliable and unambiguous quantification of phosphate molecules.

To quantify the formation of Pi, we built a calibration curve by plotting the ratio of the ^31^P NMR signal integrals of Pi and TMP as a function of known phosphate concentrations (Figure 1). A linear correlation was observed for the Pi concentrations, with an *r* ^*2*^ value of 0.998. The limits of detection (LOD) and quantification (LOQ) were calculated to be 40.3 μM and 122 μM, respectively.

**Figure 1:**
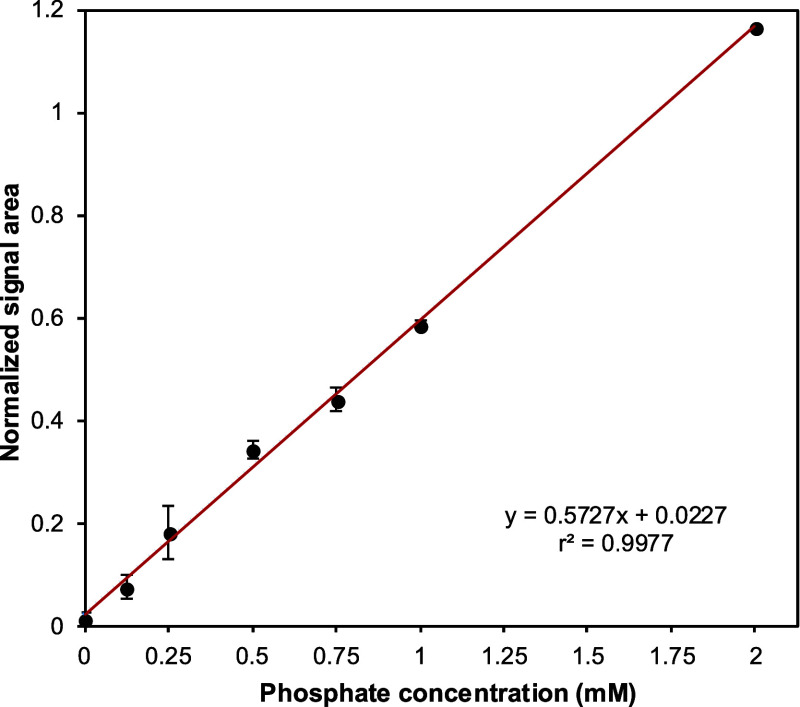
Calibration curve for phosphate quantification by ^31^P NMR. Samples containing increasing concentrations of phosphate were prepared in 40 mM Tris-HCl buffer (pH 7.5) with 2.5 mM MgCl_2_, 2% v/v DMSO, and 2.14 mM trimethyl phosphate (TMP) as internal standard. Data represents the average of experiments performed in triplicate.

### 3.2 Abl ATPase Activity Discovery

Employing the full-length Abl kinase and quantitative one-dimensional ^31^P NMR spectroscopy, we monitored changes in the NMR signals of ATP, ADP, and Pi. The quantitative protocol was adapted from Lajoie *et al*.^27^ and used to evaluate the consumption of ATP and the formation of ADP and Pi. The addition of 1.27 μM Abl kinase into a system containing 1.6 mM ATP induced ATP hydrolysis, as indicated by a decrease in the β-ATP signal (−21.6 ppm) and an increase in the Pi signal (2.5 ppm), as well as changes in the α and β phosphate groups of ADP, as observed in the spectra (Figure 2, red colored spectrum). The conversion of ATP into ADP was monitored using TMP as an internal standard to calibrate ADP and ATP NMR signals and quantify their concentrations. ATP hydrolysis was not observed in a system lacking Abl (Figure 2, blue spectrum).

**Figure 2:**
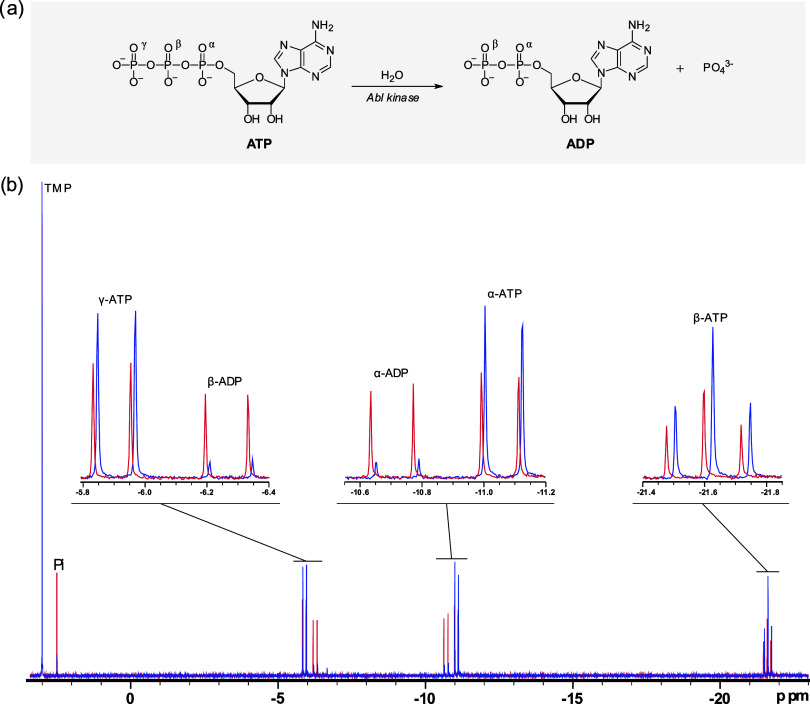
Monitoring ATP hydrolysis by quantitative ^31^P NMR. (a) Schematic representation of ATP hydrolysis. (b) ^31^P NMR spectra. The reaction medium consisted of 40 mM Tris-HCl buffer (pH 7.5), 2.5 mM magnesium chloride, and 2% v/v DMSO. The red spectrum corresponds to the reaction medium containing 1.27 μM Abl enzyme, while the blue spectrum represents the control medium without enzyme, showing no reaction. Reactions were initiated by adding ATP to a final concentration of 1.6 mM, incubated for 2 h, and then quenched with EDTA prior to ^31^P NMR analysis.

Control experiments confirmed the specificity of the Abl ATPase activity. In Abl’s absence, no spontaneous ATP conversion was observed even after 17 h of incubation. This can be visualized in the NMR spectra, where the ATP signals intensity were conserved (Supporting Information Section 2–ATP Stability Under the Assay Condition). Additionally, we investigate the possible contamination of the reaction solution with spurious phosphatases in the purified Abl samples, as such enzymes could trigger the Abl dephosphorylation and, consequently, the formation of Pi by alternative routes. Two distinct methodologies were employed to ensure the absence of phosphatase contamination. Initially, an SDS-PAGE analysis of the Abl samples was performed, and no additional bands were detected beyond those expected for the molecular weight of Abl kinase. Subsequently, *para*-nitrophenyl phosphate (pNPP), a known phosphatase substrate, was added to the samples. The hydrolysis product, *para*-nitrophenol, exhibits a yellow coloration, which can be monitored by UV–vis spectroscopy, in contrast to the colorless pNPP.^35,36^ When applied to the Abl sample, no hydrolysis of pNPP was observed. These findings demonstrate that the purified Abl kinase was free from phosphatase contamination (Supporting Information Section 3—Phosphatase Contamination Assay to Confirm Purity of Abl Kinase Preparation).

ATPase activity has been identified in a few kinases, such as Src,^37^ cAMP-dependent protein kinase,^38^ extracellular signal-regulated kinase 2 (ERK2),^39^ and protein kinase C (PKC).^40^ In the study by Rominger and colleagues,^41^ it was observed that MEK lacks detectable ATPase activity unless phosphorylated by upstream Raf kinases. Upon phosphorylation, MEK catalyzes robust ATP hydrolysis even in the absence of its substrate, ERK, indicating an intrinsic ATPase activity under specific activation conditions. This observation suggests an additional regulatory layer in MEK function and contributes to the limited but growing body of evidence that some kinases may display enzymatic activities beyond their canonical role in substrate phosphorylation. Despite sporadic reports in the literature, this area remains largely underexplored.

### 3.3 Abl ATPase Activity as a Proxy for Kinase Activity

The observed linear correlation between the initial velocity of phosphate formation and the Abl concentration (Figure 3a) is itself diagnostic of saturating substrate conditions. This interpretation is supported by the fact that the ATP concentration used (1.6 mM) was approximately 30-fold higher than its *K*_m_ value of 43.6 μM.^147^ As per the Michaelis–Menten equation, *v*_0_ = *k*_cat_[E]_t_([S]/(*K*_m_ + [S])), when [S] ≫ *K*_m_, the term [S]/(*K*_m_ + [S]) approaches unity, simplifying the relationship to v_0_ ≈ *k*_cat_[E]_t_. Under these conditions, the velocity depends linearly on the enzyme concentration, and the slope of this relationship yields the *k*_cat_. Our data demonstrate this linear dependence, showing that we are measuring the maximal catalytic rate (*v*_max_) for each enzyme concentration. Consequently, the ATPase activity of Abl can be reliably monitored under these saturating conditions, making it a valid system for inhibitor screening.

**Figure 3:**
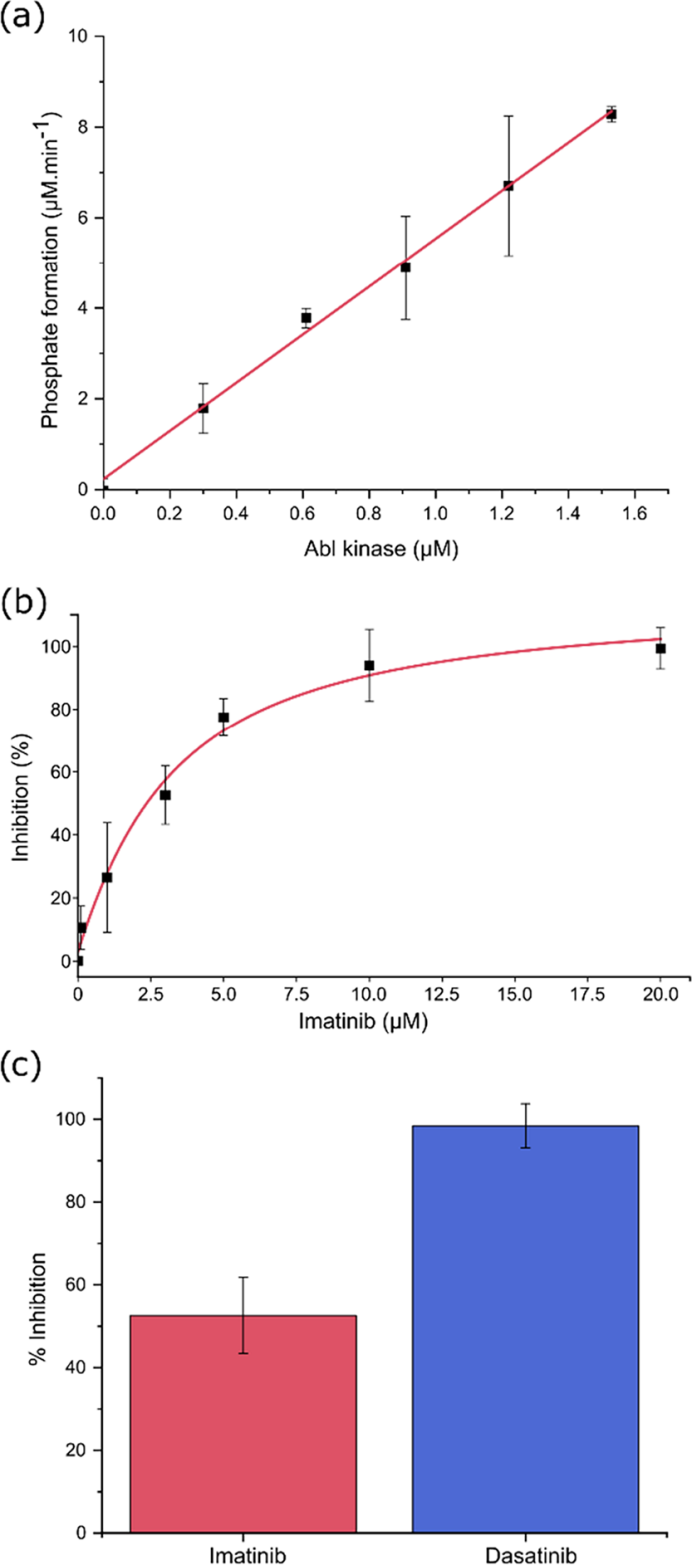
Development of an Abl assay based on ATPase activity. (a) Kinetic characterization of Abl ATPase activity by ^1^D ^31^P NMR. Reactions were carried out in 40 mM Tris-HCl buffer (pH 7.5), containing 2.5 mM MgCl_2_ and 2% v/v DMSO, initiated by the addition of ATP (final concentration: 2.58 mM). Abl was added to the reaction medium, which was incubated at 20 °C for 2 h and then quenched with EDTA. Reaction progress was monitored by ^1^D ^31^P NMR, using TMP (final concentration: 2.14 mM) as an internal standard. (b) Effect of imatinib concentration on Abl activity, assessed by ^1^D ^31^P NMR under identical buffer conditions. Abl (1.27 μM) was incubated with ATP (1.61 mM) at 20 °C for 2 h before quenching with EDTA and NMR analysis. TMP (2.14 mM) served as an internal reference. (c) Comparison of imatinib and dasatinib (3 μM each) on Abl activity. Reactions were performed as described above, with TMP (2.14 mM) as internal standard.

From these data, the turnover number (*k*_cat_) of (5.3 ± 0.2) min^–1^ (8.8 × 10^–2^ s^–1^) was obtained. Despite being significantly smaller than an ideal substrate, such as abltide (a peptide optimized for Abl kinase described by Songyang *et al*.^42^), we were able to use this reaction to measure ATP consumption as a feature for the assay. Compared to the kinase domain turnover number, we observed that the ATP hydrolysis rate is approximately 80 times lower than that reported for abltide.^43^ Nevertheless, this was expected, as it is a background reaction. The observation is consistent with previous studies on the ATPase activity of other kinases, which have generally been found to be much slower than their phosphotransferase activity.^37,44^

Comparing the ATPase activity with that of other kinases, such as the rabbit skeletal muscle phosphorylase kinase, this activity occurs at approximately 0.2% of the rate of its phosphoryl transfer activity and is three times slower than its autophosphorylation activity.^45^ Another example is the ATPase activity of Protein Kinase C (PKC) isoforms found in rat brains.^40^ Under assay conditions, the ATPase activity of the purified PKC-α and PKC-γ isoforms was 4.7% and 2.6% of the kinase activity, respectively. PKC-α hydrolyzed ATP at (0.085 ± 0.020) pmol·min^–1^, while its kinase activity was (1.80 ± 0.08) pmol·min^–1^. Similarly, PKC-γ exhibited ATP hydrolysis rates of 0.061 ± 0.018 pmol·min^–1^ and kinase activity of (2.34 ± 0.27) pmol·min^–1^.^40^ Our data align with observations for other protein kinases, corroborating ATPase activity as a background reaction. This lower level of ATP hydrolysis prevents unnecessary substrate consumption while maintaining the primary physiological function of the kinase.^44^

ATP hydrolysis by PKC occurs at the active site, as demonstrated by Ward and O’Brian.^40^ Their findings indicate that both ATPase and catalytic activities occur at the orthosteric site, supported by apparent ATP *K*_m_ values observed across different isoforms. The link between ATPase and kinase activity inhibition was confirmed for hexokinases through a systematic assessment of both activities in the presence of various inhibitors, clarifying the relationship between these enzymatic functions.^46^ For instance, 3 mM *N*-(*m*-nitrobenzoyl)-d-glucosamine inhibited 51.8% of kinase activity in the presence of the substrate, whereas in its absence, it inhibited 80.5% of ATPase activity. We applied a similar approach to assess the Abl ATPase activity in the presence of kinase inhibitors. We tested it using imatinib and dasatinib, which are competitive inhibitors targeting the ATP binding site of Abl.^47^ We hypothesized that these inhibitors could also suppress ATPase activity, reflecting their ability to inhibit kinase activity.

The enzyme inhibition data for imatinib against Abl kinase were analyzed using the Morrison tight-binding equation. This approach was necessitated by the experimental conditions, where the total enzyme concentration ([E]_T_) was not negligible compared to the inhibitor concentration ([I]_T_), thereby invalidating the assumptions underlying classical Michaelis–Menten or Hill approximations.^22,23^ The Morrison formalism accounts for the significant depletion of the free inhibitor by the enzyme, providing a more accurate determination of the dissociation constant under these conditions. From this analysis, a *K*_d_ value of 67 nM was determined. This result is near to reported values in the literature 8 nM^48^ and 26 nM.^49^

This experimentally derived *K*_d_ of 67 nM occupies an intermediate position within the range of values reported in the literature, which spans from 3.8 nM to 140 nM. A plausible explanation for this intermediate value lies in the phosphorylation status of Abl kinase during the assay.^50^ The present study measured ATPase activity under kinetic conditions in the presence of ATP. Consequently, the enzyme, initially in a dephosphorylated state, may be progressively phosphorylated throughout the reaction course. Given that Manley et al.^51^ demonstrated an influence of phosphorylation status on imatinib affinity—reporting a *K*_d_ of 3.8 nM for the nonphosphorylated form and 141 nM for the phosphorylated form—the observed value of 67 nM likely represents a population-weighted average. It reflects the dynamic equilibrium between the high-affinity (dephosphorylated) and low-affinity (phosphorylated) states of Abl kinase present in the reaction mixture, thereby reconciling our result with the existing literature.

A comparative analysis between imatinib and dasatinib at an equimolar concentration of 3 μM demonstrated different inhibitory profiles (Figure 3c): while imatinib inhibited approximately 50% of the enzymatic activity, dasatinib achieved complete inhibition under the same conditions. This pronounced difference is consistent with the known higher binding affinity of dasatinib for Abl kinase, reflecting its more potent inhibitory capacity.^52^

It is important to consider the specific limitations of the ATPase-based assay employed in this study. This method detects inhibition by measuring the decrease in the intrinsic ATP hydrolysis rate of Abl. Consequently, its applicability is inherently linked to the mechanism of action of the inhibitor. The assay is expected to be effective for compounds that directly compete with ATP binding (Type I and II inhibitors) or allosteric inhibitors that stabilize an inactive conformation, thereby impairing the catalytic apparatus necessary for phosphate transfer. However, this approach may fail to identify certain classes of inhibitors. For instance, compounds that bind exclusively to the substrate peptide-binding site without impeding ATP binding or the nucleophilic attack by water would likely not inhibit the observed ATPase activity. Similarly, allosteric inhibitors that do not lock the enzyme in a specific conformation or otherwise affect the catalytic steps of ATP binding and hydrolysis may also remain undetected. Therefore, while this ATPase assay provides a valuable and direct functional readout for a specific subset of kinase inhibitors, it is not a universal screening method, and its results should be interpreted within this mechanistic context.

The usage of ^31^P NMR is relatively expensive, and its accessibility may be limited in some settings. However, the discovery of Abl ATPase activity opens new opportunities to combine this finding with other established methods for measuring inorganic phosphate (Pi), potentially simplifying and enhancing current analytical pipelines. Among these alternative methods, those based on the molybdenum blue reaction are particularly promising. This approach, which relies on forming a phosphomolybdenum blue complex, is highly sensitive and has been widely employed for the determination of orthophosphate in environmental samples. This strategy stands out for its high sensitivity, operational simplicity, and compatibility with spectrophotometric analyses in the visible region.^53^

Also, electroanalytical methods are another potential strategy that could be coupled with ATPase activity. Sensors based on modified electrodes, metal complexes, and supramolecular systems may enable selective phosphate recognition, offering stability and reducing interference compared to optical techniques, while also facilitating miniaturization, real-time analysis, and portability.^54^ Modulating the Pi chemical equilibrium to promote strong binding to hard metal cations has been explored as a means to develop sensors for the rapid, simple, and reliable detection of phosphate species, such as those based on europium, terbium, and copper complexes.^55^ Finally, ion chromatography, renowned for its high sensitivity, is a powerful technique for phosphate analysis, proving to be another efficient approach.^56,57^

## 4 Conclusions

Here, we show the kinetic profile of Abl’s intrinsic ATPase activity, which led to the development of an ATPase activity-based assay for Abl kinase. Although the ATPase activity observed for Abl is significantly slower than its canonical phosphotransferase function, it uncovers a noncanonical enzymatic behavior with potential mechanistic and analytical implications. Reports of similar basal ATPase activities in other kinases, such as SRC, PKC, and ERK2, suggest that this may be a conserved yet underexplored feature of the kinase superfamily. It is plausible that such residual ATP hydrolysis reflects intrinsic conformational fluctuations at the active site—possibly linked to autoinhibition dynamics or catalytic resetting. From a practical perspective, we show that this ATPase activity is inhibited by ATP-competitive drugs, validating its use as a proxy for kinase inhibition. This enables the development of substrate-free assays that dispense with peptides, other enzymes, or radiolabels, offering a simplified and scalable strategy for kinase inhibitor screening. Thus, the discovery of Abl’s intrinsic ATPase activity not only expands our biochemical understanding of this key therapeutic target but also provides a foundation for novel assay formats with translational utility.

## Supplementary Material



## Notes

Experimental details regarding protein expression and purification, ATP stability assays, and tests for phosphatase activity detection (PDF)

## Abbreviations

Abl: Abelson kinase
ADP: adenosine diphosphate
ATP: adenosine triphosphate
ALL: acute lymphoblastic leukemia
Bcr: breakpoint cluster region
CML: chronic Myeloid leukemia
DMSO: dimethyl sulfoxide
EDTA: ethylenediaminetetraacetic acid
ERK2: extracellular signal-regulated kinase 2
*K*_d_: dissociation constant
LOD: limit of detection
LOQ: limit of quantification
MEK: mitogen-activated protein kinase
NMR: nuclear magnetic resonance
pNPP: *para*-nitrophenyl phosphate
PKC: protein kinase C
Pi: inorganic phosphate
SDS-PAGE: sodium dodecyl sulfate polyacrylamide gel electrophoresis
SH2: Src homology 2 domain
SH3: Src homology 3 domain
SRC: proto-oncogene tyrosine-protein kinase
TMP: trimethyl phosphate
UV–vis: ultraviolet–visible spectroscopy
