# Supplementary material for: The Discovery of Abl Kinase ATPase Activity and Its Implications in the Development of Straightforward Assays
Source: ACS Omega. 2025 Nov 12;10(46):56282–9. doi: 10.1021/acsomega.5c08176 (PMC12658686; doi:10.1021/acsomega.5c08176)
Supplement: Supplementary file 1 [file ao5c08176_si_001.pdf]

Supporting Information

The discovery of Abl kinase ATPase activity and its implications in the development of straightforward assays

Diego Magno Martins[+1], Philipe Oliveira Fernandes[+2], Lucas Almeida Vieira[1], Tiago Antônio Brandão[1], and Adolfo Henrique Moraes[1,3*]

[1]Departamento de Química, Instituto de Ciências Exatas, Universidade Federal de Minas Gerais - UFMG, 31270-901, Belo Horizonte - MG Brazil

[2]Departamento de Produtos Farmacêuticos, Faculdade de Farmácia, Universidade Federal de Minas Gerais - UFMG, 31270-901, Belo Horizonte - MG Brazil

[3]Laboratório de Ressonância Magnética de Alta Resolução - LAREMAR, Universidade Federal de Minas Gerais - UFMG, 31270-901, Belo Horizonte - MG Brazil

[+] Those authors have equally contributed to this work

**Corresponding author: adolfohmoraes@ufmg.com*

1. Protein expression and purification

1.1. Yersinia tyrosine phosphatase (YopH)

The Yop51*Δ162 plasmid was transformed into *Escherichia coli* BL21(DE3) cells by heat shock. A 200 µL aliquot of transformed cells was used to inoculate 20 mL of liquid LB medium supplemented with 20 µL of ampicillin (100 mg.mL^{-1}). This pre-inoculum was incubated at 37 °C for 16 hours with shaking at 300 rpm. Subsequently, the culture was transferred to 1 L of liquid LB medium containing 100 µg. mL ampicillin. The culture was incubated at 37 °C with shaking at 300 RPM until it reached an optical density at 600 nm (OD_{600}) of 0.7. Protein expression was induced by adding isopropyl β-D-1-thiogalactopyranoside (IPTG) to a final concentration of 400 µM. The culture was then incubated at 18 °C with shaking at 180 RPM for 16 hours. Cells were harvested by centrifugation at 4000 rpm for 45 minutes at 4 °C. The resulting bacterial pellet was collected and the supernatant discarded, and the pellet was subsequently stored at – 20 °C until further use.

Protein purification was performed as previously described[1]. The bacterial pellet containing the recombinant protein was resuspended in YopH/A buffer (100 mM acetic acid, pH 5.7, 100 mM NaCl, and 1 mM EDTA) at 0.25–0.35 g of cells per mL of buffer. Cell lysis was carried out using a high-intensity ultrasonic processor (750 W) at 35% amplitude. The sonication protocol consisted of two cycles, each comprising four 20-second on, 59-second off pulses, with a 3-minute interval between cycles. Throughout the procedure, the sample was maintained on ice. Following sonication, the lysate was centrifuged at 12,000 rpm for 40 minutes at 4 °C. The resulting supernatant was filtered and immediately used for subsequent purification steps.

Protein purification was conducted using an ÄKTA™ Start FPLC system equipped with a 280 nm UV detector. HiTrap™ SP HP (5 mL) and HiTrap™ Q HP (5 mL) ion exchange chromatography columns were initially equilibrated with YopH/A buffer. The clarified lysate was loaded onto the HiTrap SP HP column at a flow rate of 1.5 mL·min^{-1}. The column was washed with YopH/A buffer until the absorbance at 280 nm reached the baseline (approximately 8 column volumes, CV). Subsequently, the HiTrap Q HP column was connected in tandem with the SP column, and protein elution was performed using a linear ionic strength gradient by increasing the fraction of YopH/B buffer (100 mM acetic

acid, pH 5.7, 500 mM NaCl, and 1 mM EDTA) from 0 to 100%. This step was carried out at a flow rate of 2.0 mL·min^{-1}.

To prevent enzyme precipitation, the buffer was exchanged for YopH/C buffer (20 mM Tris-HCl, pH 6.5, 100 mM NaCl), a condition that enhances enzyme stability. The sample was concentrated using ultrafiltration with a Vivaspin™ 20 concentrator (GE Healthcare) to a final volume of approximately 2 mL. Subsequently, 15 mL of YopH/C buffer was added, and the concentration step was repeated to ensure thorough buffer exchange. This process was carried out three times to guarantee the complete removal of the previous buffer. Finally, glycerol was added to a final concentration of 20% (v/v). The samples were then frozen and stored at –20 °C.

The recombinant Yop51*Δ162 (33,5 KDa) enzyme was purified in a single chromatographic step using ion-exchange chromatography (Figure S1). Protein concentrations were assessed by UV at 280 nm (Cary 50, Variant), using the molar extinction coefficient 15,930 M^{-1}.cm^{-1} obtained from the ProtParam server (https://web.expasy.org/protparam/).

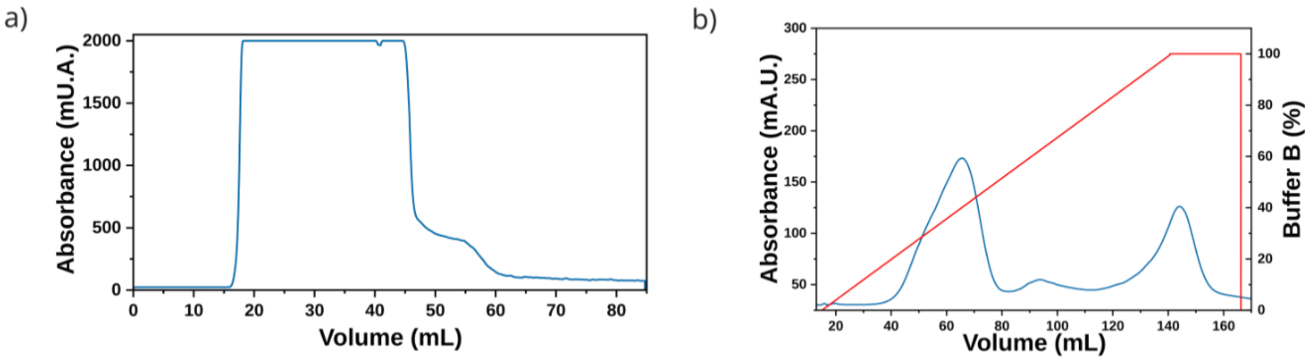

Supplementary Figure S1. Chromatographic profile of Yop51*Δ162: a) Chromatogram of the sample obtained using a HiTrap™ SP HP column (5 mL) equilibrated with YopH/A buffer (100 mM acetic acid/acetate, pH 5.7, 100 mM NaCl, and 1 mM EDTA), at a flow rate of 1.5 mL/min; b) Chromatogram of the sample purified using connected HiTrap™ SP HP (5 mL) and HiTrap™ Q HP (5 mL) columns, with a linear gradient up to 100% of YopH/B buffer (500 mM acetic acid/acetate, pH 5.7, 100 mM NaCl, and 1 mM EDTA). For both chromatographic profiles, absorbance at 280 nm (in mAU) was plotted as a function of the elution volume (in mL).

The enzymatic activity of YopH was determined by monitoring the hydrolysis of p-nitrophenyl phosphate (pNPP) to p-nitrophenol (pNP), which has a molar extinction coefficient of 18,000 $M^{-1} \cdot cm^{-1}$ at 405 nm. Absorbance measurements were performed using a Varian Cary 50 spectrophotometer, operated with CaryWinUV software and connected to a Polyscience 9106 thermostatic bath (±0.1 °C precision). All reactions were performed at 30 ± 0.1 °C in 1.4 mL quartz cuvettes containing 1.0 mL of reaction solution. Assays were conducted in 100 mM acetic acid/acetate buffer (pH 5.5) supplemented with 1.0 mM EDTA. Reactions were initiated by adding the enzyme using an add-mixer. The final enzyme concentration was 4.49 nM, and substrate concentrations were maintained below the Michaelis constant (K_m) to ensure kinetic consistency. To enable comparison with the study by Zhang *et al.* (1992)[2], the ionic strength of the reaction medium was adjusted to 0.15 M with NaCl.

YopH is a phosphatase that hydrolyzes pNPP, and the reaction rate serves as a quality control parameter for the purified enzyme. The reaction velocity was determined by monitoring the increase in absorbance at 405 nm, corresponding to the formation of pNP (Figure S2). The catalytic efficiency (k_{cat}/K_m) was determined as 158.6 $s^{-1}.mM^{-1}$ (1.6×10^5 $s^{-1} \cdot M^{-1}$), $^-$compared to a reported value of 400 $s^{-1} \cdot mM^{-1}$ (4.0×10^5 $s^{-1} \cdot M^{-1}$) (Figure S2). Although lower, the measured value remains within the same order of magnitude, indicating that the recombinant protein retains catalytic activity comparable to that reported in the literature and can be used in the following processes.

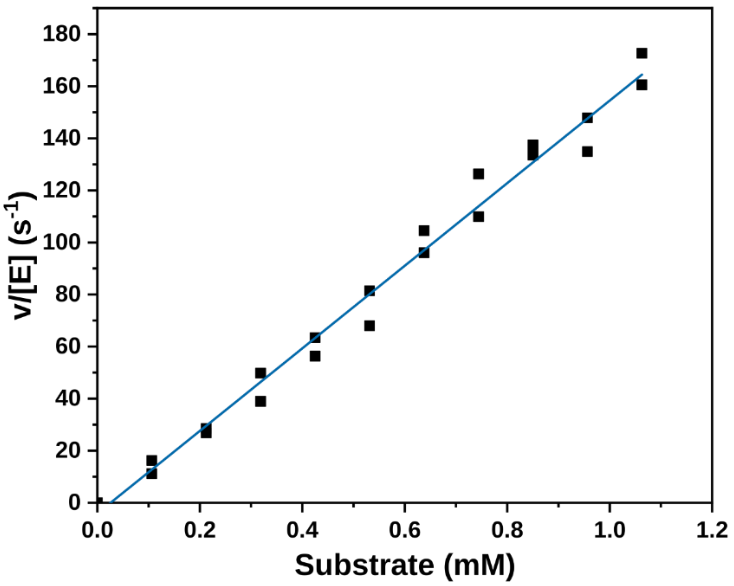

Supplementary Figure S2. Plot of the ratio between initial reaction rate and enzyme concentration as a function of substrate concentration. Kinetic profile of YopH-catalyzed pNPP hydrolysis at pH 5.5, ionic strength 0.15 M, and 30.0 ± 0.1 °C, with an enzyme concentration of 4.49 nM.

1.2. Abelson Kinase (Abl)

The plasmid encoding the Abl kinase catalytic and regulatory domains SH2 and SH3 (residues 83–534 according to Abl 1b numbering), fused to an N-terminal His_6-tag and a PreScission protease cleavage site (GE Healthcare), was kindly provided by Professor Dr. Stephan Grzesiek (University of Basel, Switzerland). Abl was co-expressed with protein tyrosine phosphatase 1B (residues 1–283) under the control of the T7 promoter in *Escherichia coli* BL21(DE3) cells[3]. Protein expression was performed following the same protocol used for YopH, with two modifications: streptomycin was used as the antibiotic (50 $\mu g \cdot mL^{-1}$ final concentration), and expression was induced with 200 µM IPTG. Five grams of cell pellet were resuspended in 35 mL of lysis buffer (50 mM Tris-HCl, pH 8.0, 500 mM NaCl, 10 mM imidazole, 5% v/v glycerol, and 3.0 mM dithiothreitol — DTT). Cell lysis was carried out by sonication as previously described for YopH.

Abl purification was performed in three chromatographic steps (Figure S3) using the same ÄKTA™ Start system described previously, following the protocol reported by

Sonti et al. (2018)[4], with the replacement of tris(2-carboxyethyl)phosphine (TCEP) with DTT in the purification buffers. In the first purification step, nickel-affinity chromatography was performed using a HisTrap™ HP 5 mL column pre-equilibrated with AblNi/A /A buffer (50 mM Tris-HCl, pH 8.0, 200 mM NaCl, 10 mM imidazole, 5% v/v glycerol, and 3 mM DTT). The clarified sample was loaded onto the column at a flow rate of 1.5 mL·min^{-1}, followed by washing with 10 column volumes (CV) of AblNi/A buffer. Protein elution was performed using a linear imidazole gradient by controlled addition of AblNi/B /B buffer (50 mM Tris-HCl, pH 8.0, 200 mM NaCl, 200 mM imidazole, 3 mM DTT, and 5% v/v glycerol), at a flow rate of 2.0 mL·min^{-1}. Fractions containing the target protein were pooled and concentrated to approximately 2 mL using a Vivaspin™ 20 centrifugal concentrator (GE Healthcare). The concentrated sample was then supplemented with 15 mL of AblNi/A buffer and 500 µL of YopH solution (150 µM final concentration) and incubated overnight (~16 hours) at 4 °C.

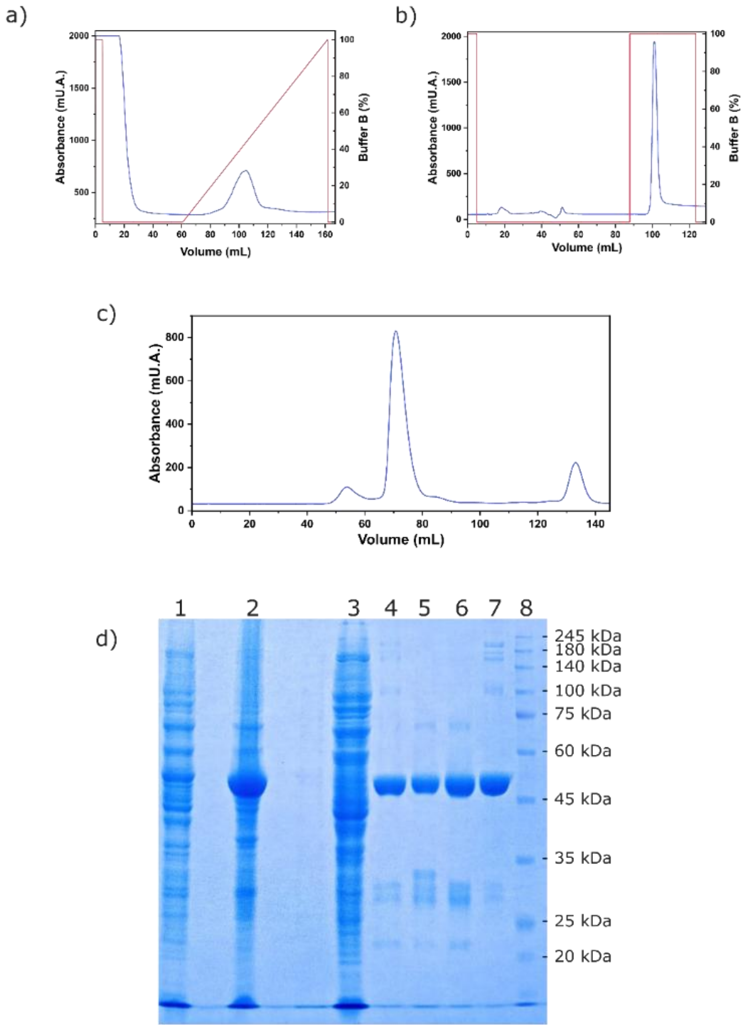

Supplementary Figure S3. Chromatographic and electrophoretic profiles of Abl kinase:(a) Chromatogram from nickel-affinity purification using a HisTrap™ HP 5 mL column, eluted at 2.0 mL/min with buffer AblNi/A (50 mM Tris-HCl, pH 8.0, 500 mM NaCl, 5% v/v glycerol, and 3 mM dithiothreitol), employing a linear imidazole gradient from 10 to 200 mM. The red line indicates the percentage of buffer B; (b) Chromatogram from a HisTrap™ HP 5 mL column following equilibration with 15 column volumes of buffer AblNi/A. Abl was eluted under isocratic conditions using buffer containing 200 mM imidazole; (c) Chromatogram from size-exclusion chromatography using a HiLoad 16/60 Superdex 75 prep grade column (GE Healthcare), equilibrated with 50 mM Tris-HCl, pH 8.0, 200 mM NaCl, 3 mM dithiothreitol (DTT), and 5% v/v glycerol, at a flow rate of 0.8 mL/min; (d) SDS-PAGE (15%) analysis. Lane 1: total lysate; Lane 2: lysate pellet; Lane 3: flow-through first affinity; Lane 4: Abl after the first affinity chromatography step; Lane 5: Abl incubated with YopH. Lane 6: Abl after the second affinity chromatography step; Lane 7: Abl after size-exclusion chromatography; Line 8: molecular weight marker (with corresponding masses indicated).

After incubation, the Abl and YopH mixture was subjected to a second nickel-affinity chromatography step (Figure S3b). A second affinity purification was chosen instead of ion-exchange chromatography, as described in the literature[4], because the phosphatase could interfere with the proposed method. After injection into the column, 15 column volumes of buffer AblNi/A were passed through to remove YopH completely. Abl was subsequently eluted under isocratic conditions using 100% AblNi/B buffer.

In the third purification step, size-exclusion chromatography was performed by loading the concentrated sample (5 mL) onto a HiLoad 16/60 Superdex 75 prep grade column (GE Healthcare) (Figure S3c). The column had been pre-equilibrated with AblNi/C buffer (50 mM Tris-HCl, pH 8.0, 200 mM NaCl, 3 mM dithiothreitol (DTT), and 5% v/v glycerol). After sample injection, the run was carried out at a flow rate of 1.0 $mL\cdot min^{-1}$. Fractions containing the protein of interest were pooled and concentrated to a final volume of 5 mL. Glycerol was added to a final concentration of 20%. The samples were then aliquoted and stored at -20 °C. The final elution buffer from the size-exclusion purification step for the NMR experiments was 20 mM potassium phosphate, pH 8.0, 200 mM NaCl.

SDS-PAGE was performed using a vertical electrophoresis unit (LCV-10×10, Loccus) to assess the monomeric mass and purity of the samples (Figure S3d). The stacking gel contained 6% polyacrylamide/bis-acrylamide, while the separating gel contained 15% polyacrylamide/bis-acrylamide. Electrophoresis was carried out at a constant voltage of 110 V during sample migration through the stacking gel and 150 V during migration through the separating gel. For band visualization, gels were stained with a dye solution containing 0.5% (w/v) Coomassie Brilliant Blue R-250, 10% (v/v) acetic acid, and 50% (v/v) ethanol.

Protein concentration was determined by UV–visible spectroscopy at 280 nm, using an extinction coefficient of 95,230 $M^{-1}\cdot cm^{-1}$ for Abl. A ^{1}H NMR experiment was performed using a pulse sequence with water signal suppression via a Watergate block (zggpw5, Bruker pulse sequence code) to evaluate protein folding (Figure S4). The final

elution buffer from the size-exclusion purification step for the NMR experiments was 20 mM potassium phosphate, pH 8.0, 200 mM NaCl.

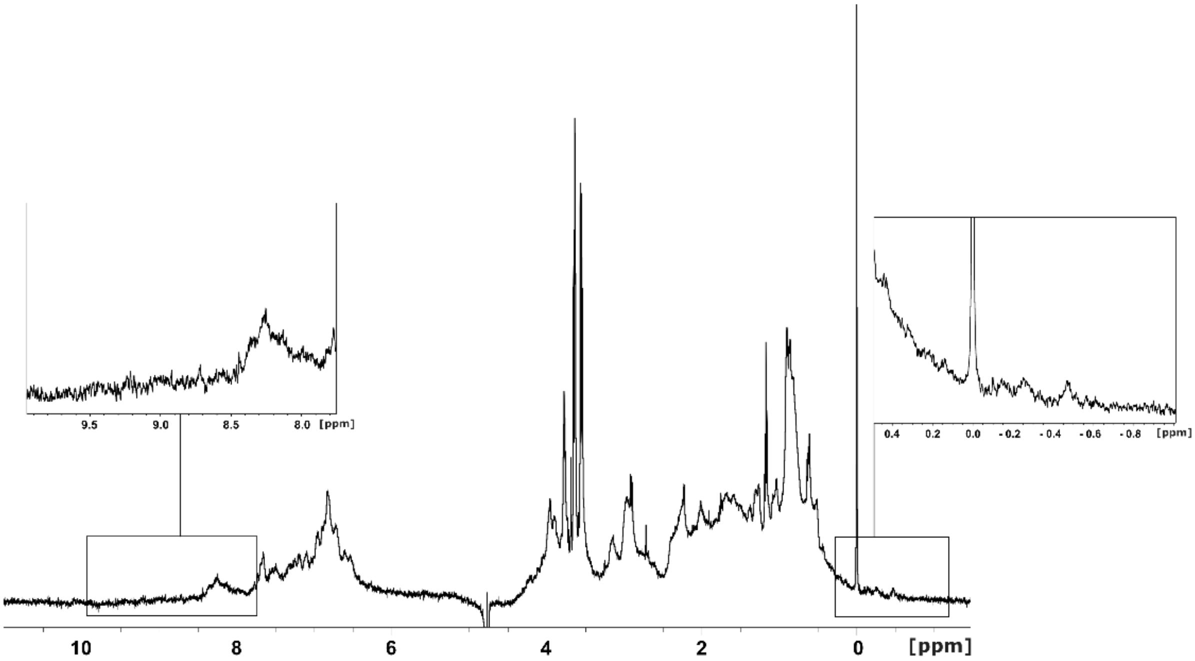

Supplementary Figure S4. 1D ^{1}H NMR spectrum of Abl kinase: the sample was prepared by solubilizing 150 µM Abl in 20 mM phosphate buffer (pH 8.0) containing 200 mM NaCl. Sodium 2,2-dimethyl-2-silapentane-5-sulfonate (DSS, 50 µM) was added as an internal chemical shift reference.

The spectrum (Figure S4) exhibits characteristic features of a folded protein. The dispersion of amide proton signals in the 6–10 ppm region indicates proper folding. Additionally, signals below 0 ppm further support this conclusion, reflecting the tendency of methyl protons to localize in highly apolar regions of the protein core, where increased electron density leads to upfield chemical shifts.[5]

2. ATP Stability Under the Assay Condition

Reactions were initiated by adding ATP to a final concentration of 1.6 mM. Three control experiments were conducted to ensure ATP stability under the reaction conditions (Figure S5). In the first control experiment, the stop solution, an EDTA solution (300 mM,

pH 13), was added to the ATP solution to simulate the quenching of the reaction in the presence of Abl. The NMR spectrum was then recorded (Figure S5b) and used as the reference for the time 0 incubation. In the second experiment (Figure S5c), ATP was added to the reaction buffer and incubated at 25 °C for 17 hours before the addition of the stop solution, allowing assessment of ATP stability under the reaction conditions. A third experiment was conducted to further investigate ATP stability in the presence of the stop solution (Figure S5d). In this case, ATP and the stop solution were added simultaneously, and the spectrum was recorded after 17 hours. In all experiments, trimethyl monophosphate (TMP) was included as an internal standard at a final concentration of 2.14 mM. All reactions were performed at 25 °C in a buffer containing 40 mM Tris-HCl (pH 7.5), 2.5 mM magnesium chloride, 2% DMSO, and 11.7% D_2O. After dilution, the final D_2O concentration was adjusted to 10% to enable frequency locking in ^{31}P NMR experiments.

Analysis of the NMR spectra (Figure S5) indicated that ATP remained stable under the tested conditions, as no significant degradation was observed. The calculated phosphate NMR integral values were consistent with those of the reference sample, confirming ATP integrity throughout the reaction timeframe.

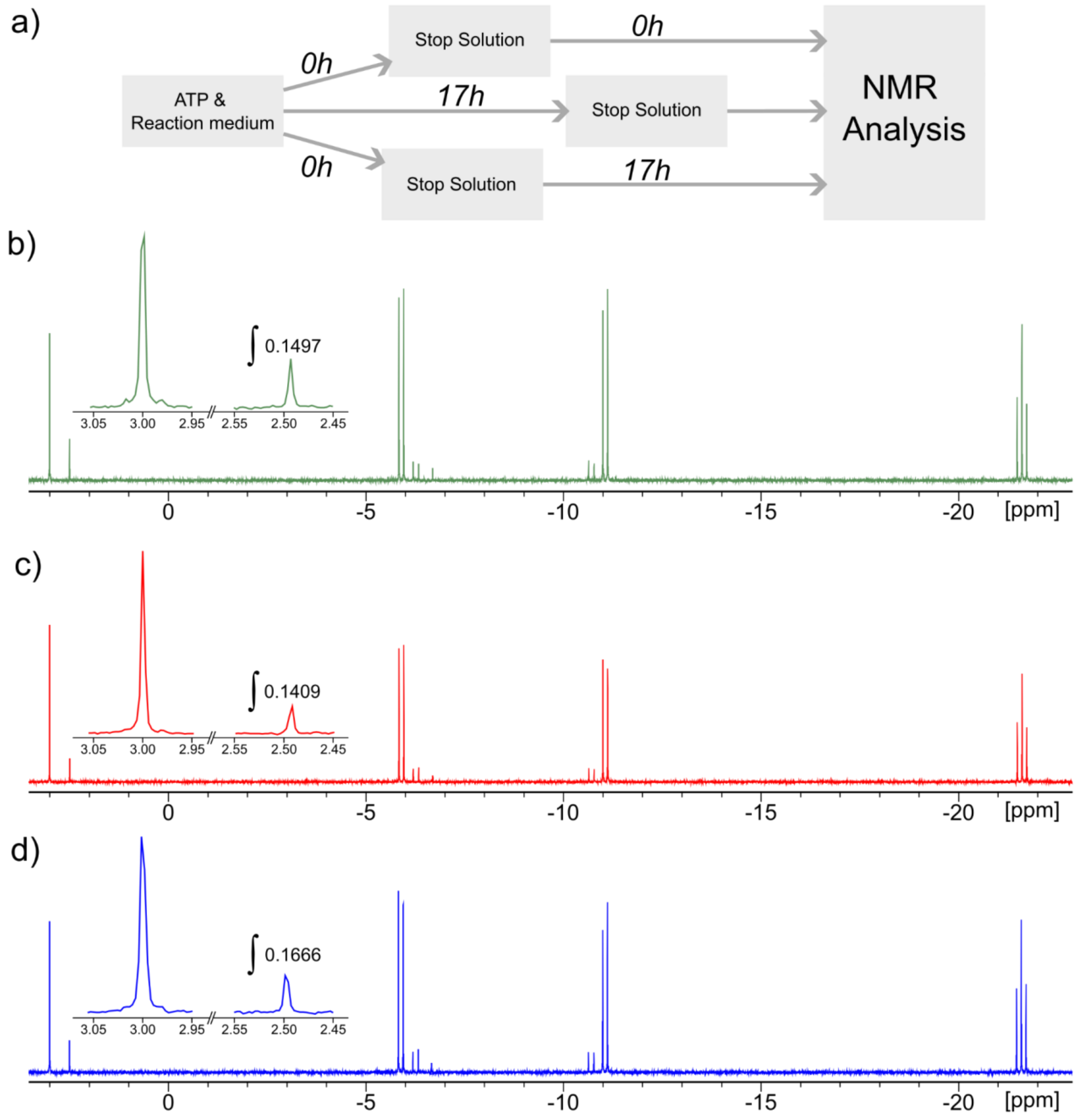

Supplementary Figure S5. ATP Stability Assay: a) Schematic representation of the three control experiments assessing ATP stability; b) ^{31}P NMR spectrum of Experiment 1 (green), in which the stop solution, an EDTA solution (30 mM, pH 13), was added to simulate the quenching of the reaction in the presence of Abl; c) ^{31}P NMR spectrum of Experiment 2 (red), where ATP remained in the reaction buffer for 17 hours before adding the stop solution; d) ^{31}P NMR spectrum of Experiment 3 (blue), in which ATP and the stop solution were added simultaneously, and the spectrum was recorded after 17 hours.

3. Phosphatase Contamination Assay to Confirm Purity of Abl Kinase Preparation

Given that Abl kinase is coexpressed with PTP1B and that YopH is introduced during one of the purification steps, potential contamination with these phosphatases could dephosphorylate autophosphorylated Abl, thereby releasing inorganic phosphate, as observed in the experiments. To confirm the absence of phosphatase contamination in the purified Abl sample, the phosphatase substrate p-nitrophenyl phosphate (pNPP) was added to the reaction solution (Figure S6).

The assay was performed in Tris-HCl buffer (40 mM, pH 7.5) containing 2.5 mM magnesium chloride, 2 % v/v DMSO and 2,1 mM pNPP. Three conditions were tested: (1) buffer only, (2) buffer with 1.27 µM Abl kinase, and (3) buffer with 37.7 nM YopH phosphatase. The reactions were monitored for two hours via UV-vis spectroscopy. Since p-nitrophenol, the hydrolysis product of pNPP, exhibits a yellow color, whereas pNPP itself is colorless, the samples were assessed for phosphatase activity (Figure S6).

Supplementary Figure S6 shows that no color change was observed in the tubes containing only buffer or Abl kinase (Figure S6 b and c), indicating that the Abl enzyme did not catalyze pNPP hydrolysis. In contrast, a yellow coloration was observed in tube 1, with a characteristic increase in the band at 420 nm (Figure S6a), containing the phosphatase YopH, confirming its expected phosphatase activity. These results demonstrate that the purified Abl kinase sample was free of phosphatases such as PTP1B and YopH, ensuring the integrity of subsequent enzymatic analyses.

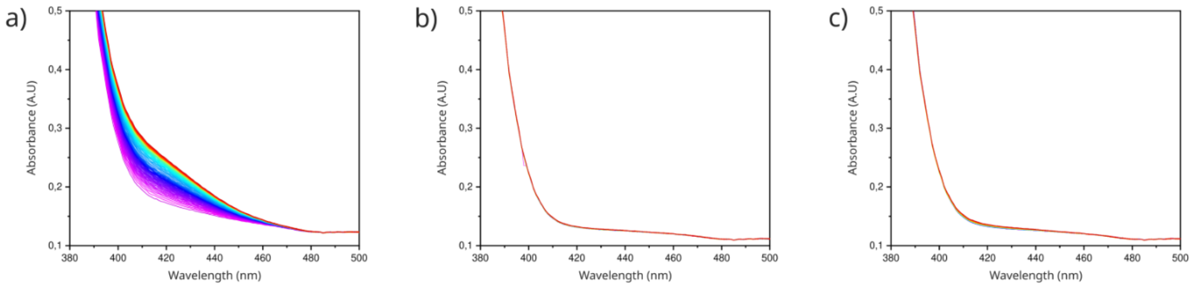

Supplementary Figure S6. Assessment of phosphatase contamination in the purified Abl sample. All three microtubes contained 40 mM Tris-HCl buffer (pH 7.5), 2.5 mM $MgCl_2$, 2% v/v DMSO, and 2.1 mM pNPP. (a) Positive control containing 37.7 nM YopH phosphatase. (b) Test sample containing purified Abl at 1.27 µM. (c) Negative control containing only buffer, without Abl or YopH. No hydrolysis of pNPP was observed in (b) or (c), confirming the absence of phosphatase contamination in the Abl preparation.

4. References

(1) Brandão, T. A. S.; Robinson, H.; Johnson, S. J.; Hengge, A. C. Impaired Acid Catalysis by Mutation of a Protein Loop Hinge Residue in a YopH Mutant Revealed by Crystal Structures. *J. Am. Chem. Soc.* **2009**, *131* (2), 778–786. https://doi.org/10.1021/ja807418b.

(2) Zhang, Z. Y.; Clemens, J. C.; Schubert, H. L.; Stuckey, J. A.; Fischer, M. W.; Hume, D. M.; Saper, M. A.; Dixon, J. E. Expression, Purification, and Physicochemical Characterization of a Recombinant Yersinia Protein Tyrosine Phosphatase. *Journal of Biological Chemistry* **1992**, *267* (33), 23759–23766. https://doi.org/10.1016/S0021-9258(18)35903-9.

(3) Skora, L.; Mestan, J.; Fabbro, D.; Jahnke, W.; Grzesiek, S. NMR Reveals the Allosteric Opening and Closing of Abelson Tyrosine Kinase by ATP-Site and Myristoyl Pocket Inhibitors. *Proceedings of the National Academy of Sciences* **2013**, *110* (47), E4437–E4445. https://doi.org/10.1073/pnas.1314712110.

(4) Sonti, R.; Hertel-Hering, I.; Lamontanara, A. J.; Hantschel, O.; Grzesiek, S. ATP Site Ligands Determine the Assembly State of the Abelson Kinase Regulatory Core via the Activation Loop Conformation. *J. Am. Chem. Soc.* **2018**, *140* (5), 1863–1869. https://doi.org/10.1021/jacs.7b12430.

(5) Barile, E.; Pellecchia, M. NMR-Based Approaches for the Identification and Optimization of Inhibitors of Protein–Protein Interactions. *Chem. Rev.* **2014**, *114* (9), 4749–4763. https://doi.org/10.1021/cr500043b.
